# Blood–Brain Barrier Disruption as a Potential Target for Therapy in Posterior Reversible Encephalopathy Syndrome: Evidence From Multimodal MRI in Rats

**DOI:** 10.3389/fneur.2019.01211

**Published:** 2019-11-26

**Authors:** Quanlai Wang, Bin Huang, Guiquan Shen, Yu Zeng, Zheng Chen, Chunqiang Lu, Alexander Lerner, Bo Gao

**Affiliations:** ^1^Department of Imaging, Zhoukou Central Hospital, Zhoukou, China; ^2^Department of Radiology, Affiliated Hospital of Guizhou Medical University, Guiyang, China; ^3^Jiangsu Key Laboratory of Molecular and Functional Imaging, Southeast University, Nanjing, China; ^4^Division of Neuroradiology, Department of Radiology, Keck School of Medicine, University of Southern California, Los Angeles, CA, United States

**Keywords:** posterior reversible encephalopathy syndrome, acute hypertension, blood–brain barrier, MRI, rat models

## Abstract

**Background:** To explore blood–brain barrier disruption in hypertensive posterior reversible encephalopathy syndrome.

**Methods:** The hypertension rat models were successfully established and scanned on 7T micro-MRI. MRI parameter maps including apparent diffusion coefficient, T1 value, and perfusion metrics such as cerebral blood volume, cerebral blood flow, mean transit time and time to peak maps, were calculated.

**Results:** The ADC values of the experimental group were higher than those of the control group both in cortical (*P* < 0.01) and subcortical (*P* < 0.05) regions. Voxel-wise analysis of ADC maps localized vasogenic edema primarily to the posterior portion of the brain. The increase in cerebral blood volume and cerebral blood flow values were found in the cortical and subcortical regions of rats with acute hypertension. No correlation was found between perfusion metrics and mean arterial pressure. The Evans blue dye content was higher in the posterior brain region than the anterior one (*P* < 0.05).

**Conclusions:** Cerebral vasogenic edema resulting from acute hypertension supports the hypothesis of posterior reversible encephalopathy syndrome as the result of blood–brain barrier disruption, which maybe the potential therapeutic target for intervention.

## Introduction

Posterior reversible encephalopathy syndrome (PRES) usually occurs following a precipitous rise of blood pressure (1). Acute hypertension may lead to persistent and severe disorder of cerebral circulation with passively or forced dilation of the cerebral arterioles, resulting in cerebral hyperperfusion, brain edema, and increased intracranial pressure (2, 3). Cerebral edema primarily in bilateral occipital and parietal lobes constitutes the characteristic radiological findings in patients with PRES (4, 5). If the clinical intervention in PRES is delayed or ineffective, severe neurological complications or even death may occur (6).

The mechanism of PRES is controversial. One of the possible inciting factors is the rapid rise in blood pressure, which exceeds the upper limit of cerebral autoregulation, and results in excessive blood flow with subsequent blood–brain barrier (BBB) breakdown. The BBB is a complex multicellular structure acting as selective barrier controlling the transport of substances between intravascular and extravascular interstitial space compartments (7). BBB breakdown allows for transgression of plasma and macromolecules from the vessels into the interstitial spaces leading to vasogenic edema (8, 9). Alternatively, severe hypertension may lead to excessive reaction of the cerebrovascular autoregulation, spasm of small cerebral vessels, and decrease in perfusion, resulting in ischemia, BBB disruption, increased vascular permeability, and brain edema (10). The mechanism of cerebral edema in PRES remains to be elucidated (11). Therefore, the most important aspect of treatment in PRES is aggressive management of blood pressure. In some studies, hypertension above the autoregulatory limit has led to BBB breakdown and vasogenic edema (12). Some studies have aimed at BBB integrity through chemical and physical therapies to achieve therapeutic effects (7), and some studies have suggested that the integrity of the BBB correlates to the outcomes in this disorder (13, 14). Therefore, preservation of the integrity of BBB is important in treatment of PRES. PRES is characterized as a rapid, dynamic, and transient process of disturbance of cerebral hemodynamics. Delayed perfusion imaging or use of antihypertensive therapy during the examination may lead to inconclusive diagnostic findings and result in hemodynamic changes (15). We hypothesized that abnormalities could be detected with diffusion weighted images (DWI) and perfusion-weighted imaging (PWI) in PRES and that “normal appearing” regions would have increased water diffusion and hyperperfusion. The aim of the present study is to investigate whether cerebral edema, hemodynamic change, and BBB disruption can be detected in rat model using 7.0 T micro-MRI and to elucidate the pathophysiological mechanism of PRES.

## Methods

### Animal Model Preparation and Procedures

The present study was approved by the Laboratory Animal Management Committee of Southeast University. All operations were performed according to the international guidelines concerning the care and treatment of experimental animals. A rat model of acute hypertension was established. Forty male Wistar rats weighting 250–300 g were randomly grouped into the experimental group (*n* = 20) and the control group (*n* = 20). Rats were introduced to anesthesia with 5% isoflurane, and anesthesia was maintained with intraperitoneal injection of pentobarbital (40 mg/kg, 2% in saline). A saline solution of Evans blue (EB) was injected via the tail vein. A vertical incision on both sides of inguen was performed in the rats. A femoral arterial catheter, which was connected to the pressure transducer and a physiological monitor, was inserted in one of the femoral arteries to measure the blood pressure. Femoral venous catheter was inserted for continuous injection of phenylephrine (PE) during MRI scanning. The dosage of PE was started at rate of 0.5 μl/min and increased by 0.5 μg/min. Another femoral venous catheter was also inserted in advance to inject gadolinium–diethylenetriaminepentaacetic acid (Gd-DTPA) before MRI scanning. The rats were then placed on the table of the MRI scanner and immobilized with a teeth bar and two ear bars. When the systolic blood pressure (SBP) reached 180 mmHg or mean arterial pressure reached 150 mmHg, the MRI scan would be initiated. The methods mentioned above are described by Euser et al. (16). During the preparation, temperature was maintained between 37 and 37.5°C with a self-regulating heating pad. After the MRI scan, the animal was quickly decapitated, and the brain was removed for histopathological examination. The control group was injected with saline solution instead of PE otherwise following the same procedure. We also record the duration of anesthesia of the rats; the same period of time after injection of anesthetics was ensured for all rats to be scanned by MRI.

### MRI Protocols

MRI was performed on 7.0 T micro-MRI scanner (Bruker PharmaScan, Germany). Anesthesia was induced and maintained by inhalation of 1.5% isoflurane (Shandong Keyuan Pharmaceutical Co., Ltd., China). The body temperature was maintained with a feedback-controlled water bath warming system (MT1025, Bruker Biospin Inc., Germany), and the respiratory rate was monitored by a monitoring unit (Model 1025, SA Instruments Inc.). A quadrature volume resonator (inner diameter, 72 mm) was used for radio frequency transmission, and a four-element surface coil array was used for signal reception. Experiments were executed with ParaVision 5.1 software. To optimize field homogeneity, a field-map-based MAPSHIM method was used for shimming. Rapid acquisition with relaxation enhancement T2-weighted sequence was acquired in the axial plane with TR/TE, 3,000/36 ms; matrix size, 256 × 256; thickness, 1 mm; field of view (FOV), 320 mm × 320 mm; slice number, 22; average, 1. DWI were acquired with TR/TE 6,250/30 ms; matrix, 128 × 128; thickness, 1 mm; FOV, 320 × 320 mm, slice number, 22; average, 2; *b* values = 100, 200, 400, 600, 800, and 1,000 s/mm^2^. For T2^*^-weighted dynamic susceptibility contrast-enhanced perfusion-weighted imaging (T2^*^-DSC-PWI), a GE-EPI sequence was used with TR/TE, 1,000/9 ms; FOV, 320 × 320 mm; matrix, 64 × 64; repetition, 200; and in-plane resolution of 0.5 × 0.5 mm. The intravenous bolus of gadodiamide (0.1 mmol/kg, 4 ml/s) was started after the 15th measurement was obtained. For T1 mapping, a RAREVTR sequence with six repetition times, TE of 11 ms, FOV of 320 × 320 mm, matrix of 128 × 128, thickness of 1 mm, slice number of 20, average 1 was used.

### BBB Permeability

After MRI scanning, the animal was perfused with phosphate buffered saline through the ascending aorta to remove the dye from the vasculature. The whole brain was removed and divided into two halves: the posterior and anterior cerebrums sections, by making a cut in a coronal plane at the level of the optic chiasm. The tissue was homogenized in 5 ml 50% trichloroacetic acid and centrifuged (4,000*g*, 10 min). After centrifugation, the supernatant was diluted three-fold with ethanol and analyzed by fluorescence spectrophotometry (620–680 nm) to determine EB content, with the data expressed as average fluorescence counts *per second* (CPS) per gram brain tissue.

### Imaging Analysis and Post-processing

Before any further processing, raw ParaVision DWI and EPI datasets were converted to 32-bit NIFTI format. The NIFTI EPI datasets were then converted back to 16-bit DICOM format using a custom-made Matlab script to ensure the original raw data range. ADC maps and T1 maps were calculated with MRI analysis plugin (https://imagej.nih.gov/ij/plugins/mri-analysis.html) in Image J (Version 1.50f; National Institutes of Health, Bethesda, USA; http://rsbweb.nih.gov/ij). The PWI images in DICOM format were processed using Perfusion Mismatch Analyzer (http://asist.umin.jp/data-e.shtml) software. After creation of time intensity curve, the bolus start time and bolus end time were determined. Time concentration curves for each pixel were generated from the time intensity curves. Arterial input function pixels were automatically selected. Quantitative maps including cerebral blood volume (CBV), cerebral blood flow (CBF), mean transit time (MTT), and time to peak (TTP) were calculated by deconvolution the tissues curves using arterial input function. The maps of CBV, CBF, MTT, and TTP were generated automatically. Region of interest (ROI) analysis was performed in the PMA software. Two ROIs corresponding to cortex and subcortical regions were selected. The ADC maps and T1 maps were coregistered to a rat template set based on the standard rat brain atlas of Paxinos and Watson using Statistical Parametric Mapping (SPM8, http://www.fil.ion.ucl.ac.uk/spm/). Then, spatial smoothness with an isotropic Gaussian kernel (FWHM = 2 voxels) was performed on the spatial transformed images. Two sample *t*-tests were performed on the ADC images and T1 images of the two groups. *P* < 0.05 was considered to be statistically significant. Multiple comparison correction was performed using AlphaSim method (http://afni.nih.gov/afni/docpdf/AlphaSim.pdf).

### Statistical Analysis

Except for voxel wise analysis, all statistical analysis was performed using SPSS software (version 18.0). All values were expressed as mean ± SD. The comparison between the groups was performed using independent sample *t*-test. Differences in tissue fluorescence between the anterior and posterior cerebrum within the same intervention group were tested with paired *t*-test. *P* < 0.05 was considered as significant.

## Results

### Arterial Pressure

**Figure 1:**
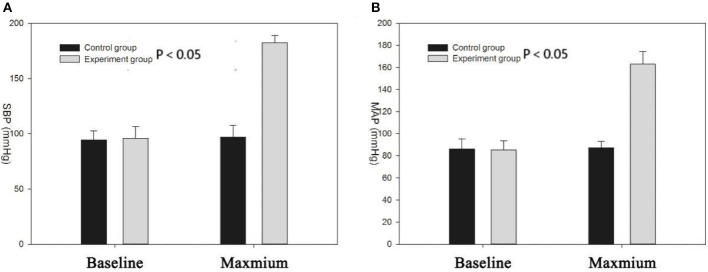
**(A)** Systolic blood pressure (SBP) of experimental group increased significantly compared with the control group (*P* < 0.05); the two groups had no significant difference in baseline blood pressure; **(B)** mean arterial pressure (MAP) of experimental group increased significantly compared with the control group (*P* < 0.05).

Figure 1 shows SBP and mean arterial pressure (MAP) values before and after PE injection in the experiment group and saline solution injection in the control group. The SBP and MAP in the experimental group were significantly higher than those of the controls (SBP, 182.16 ± 6.83 mmHg vs. 97.01 ± 10.65 mmHg; MAP: 162.91 ± 5.93 vs. 87.25 ± 11.37, *P* < 0.05). After a rapid rise in blood pressure, the rats were characterized by shortness of breath, rapid heartbeat, ocular proptosis, muscle contraction, salivation, and runny nose. There was no significant difference in the duration of anesthesia of the rats (time: 126 ± 6 min, *P* > 0.05).

### Comparison of Changes of DWI, ADC, and T1 Values

**Figure 2:**
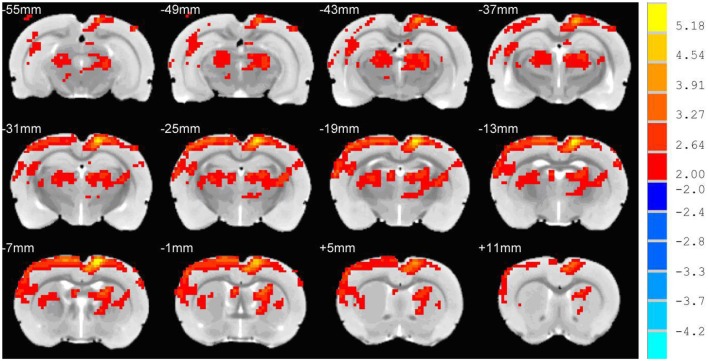
Voxel wise analysis of the ADC maps. The red and yellow region was the region of the ADC value of the experimental group, which was significantly higher than the ADC value of the control group. The cerebral edema region was mainly seen in the posterior region of the rat brain.

There were no significant abnormities on both T2 images and DWI images before and after hypertension modeling. The ADC value of the experimental group was significantly higher than that of control group both in the cortex and subcortical regions (cortex: 5.1 ± 0.49 vs. 5.48 ± 0.47, *t* = 3.291, *P* < 0.01; subcortical: 5.33 ± 0.23 vs. 5.59 ± 0.32, *t* = 2.186, *P* < 0.05). Voxel wise analysis of the ADC maps showed that the vasogenic brain edema was located in the parieto-occipital cortex, subcortical nuclei, thalamus, brain stem, and cerebellum, primarily localizing to the posterior cerebral region of the rats (Figure 2). There was no cluster showing statistical significance in voxel wise analysis of the T1 mapping.

### Comparison of PWI Parameters

**Figure 3:**
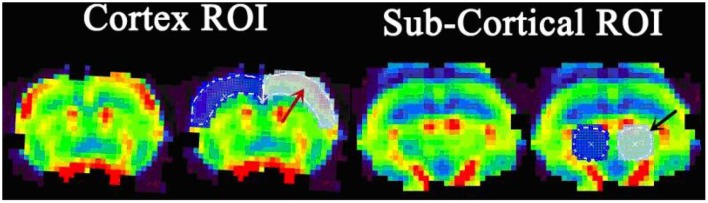
The definition of region of interest (ROI) delineated on the perfusion image. The cortex ROI (red arrow) covers the bilateral cortex of the slice near the bregma. The subcortical ROI (black arrow) covers the bilateral thalamus, located in the slice 2 mm posterior of bregma.

**Figure 4:**
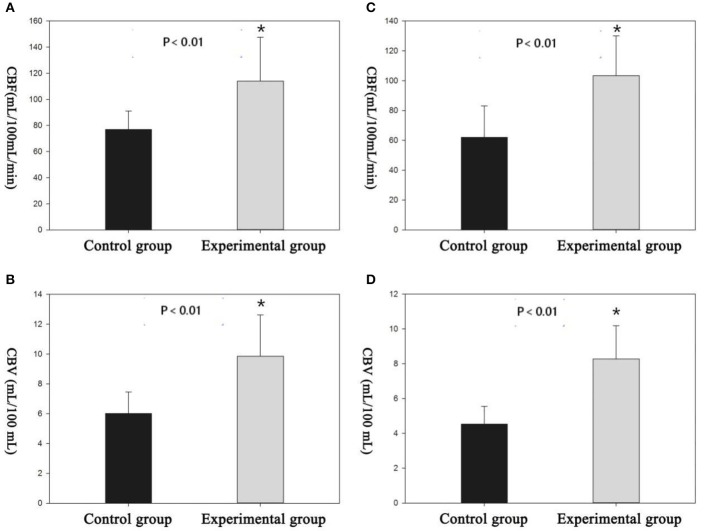
There were significant differences in perfusion parameters between the experimental group and the control group. **(A,B)** Cortical cerebral blood flow (CBF) and cerebral blood volume (CBV) measured by region of interest (ROI). **(C,D)** Subcortical CBF and CBV measured by the ROI. *Difference is statistically significant.

**Figure 5:**
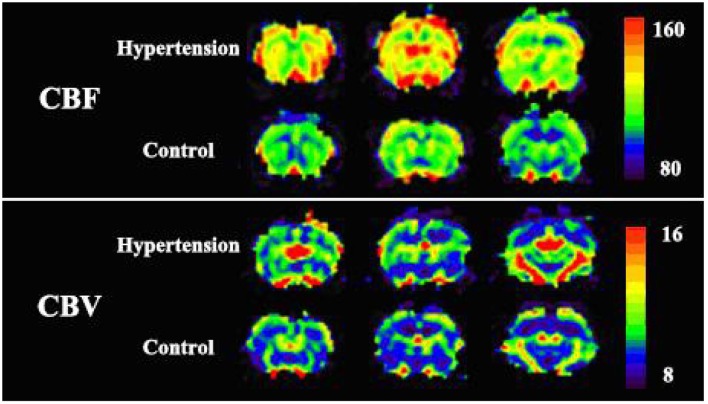
The perfusion image of two representative rats of each groups. Cortical cerebral blood flow (CBF) and cerebral blood volume (CBV) values in the experimental group were significantly higher than the values in the control group. No apparent change in mean transit time (MTT) was observed in the whole brain of the two rats.

Cortical and subcortical region values for CBF, CBV, and MTT parameters were calculated. The selection of ROI is shown in Figure 3. All rats demonstrated increased CBV (cortex: *t* = 4.319, *P* < 0.01; subcortex: *t* = 6.355, *P* < 0.01) and CBF (cortex: *t* = 3.764, *P* < 0.01; subcortex: *t* = 4.33, *P* < 0.01) values within cortex and subcortical regions (Figure 4). Figure 4 shows the PWI parameter images of two rats representing the acute hypertension group and the control group, respectively. No significant difference in MTT values was detected (Figure 5). Furthermore, no correlation was found between CBF and MAP (cortex: *r* = 0.117, *P* = 0.622; subcortex: *r* = 0.1, *P* = 0.674); and no correlation was demonstrated between CBV and MAP (cortex: *r* = 0.245, *P* = 0.297; subcortex: *r* = 0.043, *P* = 0.858).

### Blood–Brain Barrier Permeability

**Figure 6:**
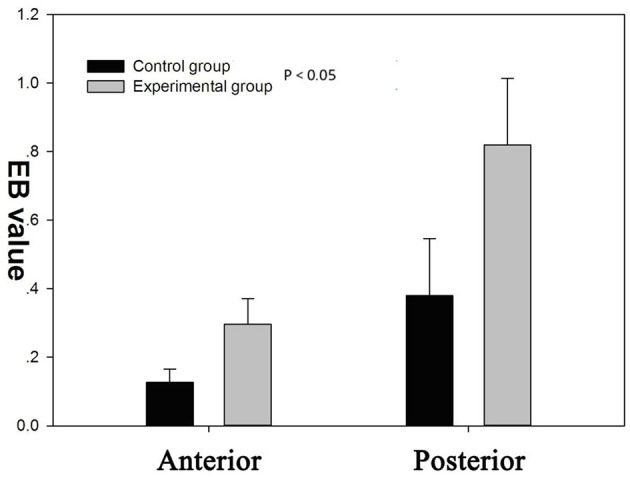
There was a significant increase of blood–brain barrier (BBB) permeability to Evans blue (EB) in both the anterior and posterior cerebrum (*P* < 0.05). BBB permeability to EB varied regionally in response to autoregulatory breakthrough, such that the increase in BBB permeability was significantly greater in the posterior brain region than anterior.

**Figure 7:**
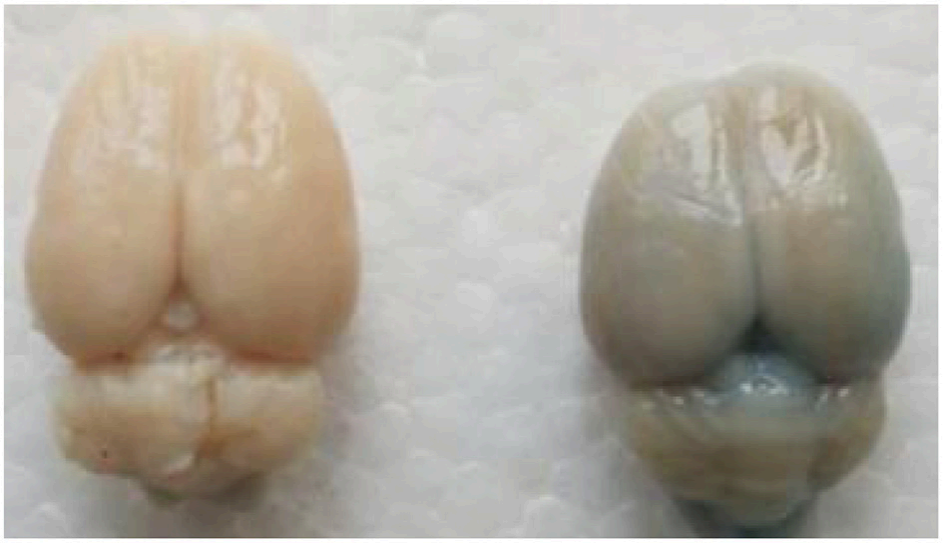
In the experimental group, Evans blue (EB) dye content was significantly higher than control group. The presence of blue-staining areas in the brain tissue in the experimental group indicates the disruption of the blood–brain barrier.

There was an increase in EB dye content (CPS/g) both in posterior and anterior cerebrum compared with control group (Figures 6, 7). In addition, the increase in the EB dye content was significantly higher in the posterior brain region than in the anterior (*P* < 0.05), as the former was more susceptible to the formation of edema.

## Discussion

Several prior studies have verified the feasibility of an acute hypertension animal model (16–20). Most of the previous studies focused on pathophysiology and rarely used imaging to evaluate the distribution of acute hypertensive cerebral edema, hemodynamic changes, and BBB disruption (21). This study investigated the distribution of cerebral edema and cerebral perfusion changes non-invasively using DWI and T2^*^-DSC and evaluated the BBB damage qualitatively using T1 mapping. MRI findings of brain edema in PRES characteristically occur in the posterior circulation areas in the parietal–occipital lobes and cerebellum, with associated T2 hyperintensity (22). In this study, no marked signal change was observed on T2WI and DWI images in the acute hypertension model. However, the ADC values were significantly higher than those of the control group with ROI analysis. The transport of albumin-bound EB through BBB accumulated in the extracellular spaces, which provided the evidence of BBB damage (23). If blood pressure is too high, it exceeds the brain's ability to regulate blood flow automatically. This leads to increased arterial and capillary pressure, resulting in a rupture of the BBB and leakage of fluid and proteins into the brain parenchyma, leading to vasogenic edema (24). Vasogenic edema on DWI may result in hypointensity or hyperintensity (due to T2 shine-through effect of vasogenic edema on DWI). Hypointensity on DWI is seen in some cases with diffusion facilitation with elevated ADC values, while cytotoxic edema shows hyperintensity with decreased ADC values (25). Moreover, the ADC images may demonstrate abnormalities that cannot be identified on T1WI or T2WI (25, 26).

The voxel-wise analysis of ADC images of the whole brain showed that brain edema was mainly located in the cortex of the posterior cerebrum. The preferential formation of edema in the posterior regions is due to the elevated BBB permeability. BBB permeability to EB varies regionally in response to autoregulatory breakthrough, such that increase in BBB permeability was significantly greater in the posterior regions than in the anterior regions (27, 28). The blue-stained area in the cerebrum, cerebellum, and other regions with EB observed staining in the experimental group were concordant with the distribution of cerebral edema on ADC maps. The anterior cerebral circulation has more autonomic receptors than the posterior circulation; consequently, cerebral autoregulation is more susceptible in the posterior vascular territory (24, 25). Such regional differences in brain regions have not been described in rats (29, 30). Furthermore, we found that these phenomena were detected in anterior cerebrum in part due to the duration of extended hypertension state over 1 h. This long-term hypertension state results in sustained hyperperfusion, which may result in decreased sympathetic nervous stimulation of the anterior circulatory vessels, causing the involvement of anterior regions in the brain. We hypothesized that these two factors may explain the lack of preference for regional changes in the posterior of brain in rats.

T1 mapping was used to compare abnormal enhancement area before and after injection of Gd-DTPA to determine whether the permeability of BBB has changed. Normally, Gd-DTPA cannot pass through BBB and is confined in the blood vessel. If there is Gd-DTPA enhancement in a certain region of the brain, this indicates that BBB is disrupted and that permeability is increased (31). However, no abnormal enhancement region was observed on the T1 mapping in this study. This may be due to the fact that we acquired the T1 image after a short delay, and a longer delay may be necessary to detect the signal changes caused by the slow accumulation of contrast agents in extracellular space (32).

In this short-term acute hypertension model, when compared to the control group, the values of CBF and CBV in the acute hypertension group increased significantly, and we found a significant increase in CBF and CBV in the cortical and subcortical areas, and the increase in CBV and CBF in the cortex was greater than that of subcortical regions. This was more evident in the posterior areas of brain, indicating that the increased perfusion primarily involved the posterior regions of the brain. This suggests that hyperperfusion is the result of cerebral vascular autoregulation dysfunction, and it has also been proved in animal experiments (33). the presence of hyperperfusion in acute hypertension confirmed that vasogenic edema appears in the early stages of this disorder due to high capillary pressure and increased BBB permeability. Some PWI studies also found that patients with hypertensive encephalopathy had hyperperfusion, and the evidence was as strong (34, 35). Decreased cerebral perfusion in edema areas has also been reported (36, 37). Sundgren et al. (38) found that microvascular perfusion in region of edema was decreased, with decrease in CBF and CBV. In addition, when blood pressure was successfully controlled, the perfusion volume in the area of edema returned to normal. However, no significant change in MTT was detected either in cortex or in subcortical regions. MTT may contribute to the degree of vasoconstriction (39). Inconspicuous MTT change did not indicate the systolic or diastolic condition of vessels, which may be due to poor sensitivity of the methods, resulting in lack of detectable MTT changes (40).

In this study, we used multimodal MR imaging to study the acute hypertension model producing results which help to elucidate the pathophysiological basis of BBB damage in PRES. This study is limited by lack of longitudinal data, and there are some limitations in our study. First, we did not record the time of hypertension in animals. Second, EB dye content would be directly proportional to CBF and to the time length from EB injection to the time when the brains were harvested. However, the results of EB dye content were not corrected by CBF and the time length from EB injection to the time when the brains were harvested; we should pay attention to these factors in the future studies. Third, Gd-DTPA enhanced-T1 failed to show any difference between the control and experimental group; therefore, in this study, our animal model may not apply to all PRES due to not all PERS having or being associated with hypertension. We did not find significant abnormities on neither T2 images nor DWI images. In the future, it is necessary to build a more reasonable and applicable PRES model. Subsequent studies using longitudinal design are needed for further investigation of PRES pathophysiology.

## Conclusion

The pattern of vasogenic cerebral edema resulting from acute hypertension in the rat model suggests that BBB disruption is an important component of PRES pathophysiology, representing a potential target for therapeutic intervention.

## Data Availability Statement

All datasets generated for this study are included in the article/supplementary material.

## Ethics Statement

This study was carried out in accordance with the recommendations of international guidelines concerning the care and treatment of experimental animals, the Institutional Animal Care and Use Committee. The protocol was approved by the Institutional Animal Care and Use Committee.

## Author Contributions

BG, GS, and AL contributed conception and design of the study. YZ contributed data collection. QW and BH contributed data processing. CL performed the statistical analysis. QW wrote the first draft of the manuscript. BH and ZC wrote sections of the manuscript. All authors contributed to manuscript revision, read, and approved the submitted version.

### Conflict of Interest

The authors declare that the research was conducted in the absence of any commercial or financial relationships that could be construed as a potential conflict of interest.
